# Bilateral Lesions Are Linked to Postoperative Regrowth in Craniofacial Fibrous Dysplasia: Alkaline Phosphatase as a Marker of Clinical Phenotype

**DOI:** 10.3390/diagnostics16030472

**Published:** 2026-02-03

**Authors:** Jiang Xue, Longping Liu, Jianyun Zhang, Yue Lou, Lisha Sun, Tiejun Li

**Affiliations:** 1 Department of Oral Pathology, Peking University School and Hospital of Stomatology, Beijing 100081, China; 2 National Center of Stomatology, National Clinical Research Center for Oral Diseases, National Engineering Research Center of Oral Biomaterials and Digital Medical Devices, Beijing 100081, China; 3 Central Laboratory, Peking University School and Hospital of Stomatology, Beijing 100081, China

**Keywords:** fibrous dysplasia, alkaline phosphatase, prognosis

## Abstract

**Objectives:** This study aimed to evaluate phenotypic associations between preoperative alkaline phosphatase (ALP) levels and clinical characteristics, and explore clinical factors associated for postoperative regrowth in craniofacial fibrous dysplasia. **Methods:** In this retrospective cohort (2003–2024), 71 surgically treated fibrous dysplasia patients were analyzed. Relationships between preoperative ALP (using age-stratified reference ranges) and key phenotypes (age at surgery, onset age, laterality, lesion type) were assessed via nonparametric tests. Associations with postoperative regrowth were assessed using Mann–Whitney U or Kruskal–Wallis tests for non-normally distributed continuous variables and χ^2^ or Fisher’s exact tests for categorical variables. **Results:** Preoperative ALP levels significantly correlated with younger surgical age (16–19 vs. ≥19 years: 244.0 vs. 107.0 U/L, *p* < 0.001), earlier onset (0–16 vs. >16 years: 114.0 vs. 83.0 U/L, *p* = 0.030), bilateral lesions (176.0 vs. 106.2 U/L, *p* = 0.006), and polyostotic subtype (polyostotic fibrous dysplasia vs. monostotic fibrous dysplasia: 162.0 vs. 87.5 U/L, *p* < 0.001). However, neither ALP levels (*p* = 0.061) nor abnormal ALP rates (*p* = 0.090) predicted regrowth. Crucially, bilateral lesions were significantly associated with regrowth (83.3% (5/6) vs. 21.5% (14/65); *p* = 0.005). The overall regrowth rate was 8.5% (6/71). **Conclusions:** Bilateral lesions demonstrate significant association with postoperative regrowth risk, potentially guiding surveillance intensity. ALP correlates with phenotypic burden but shows limited prognostic utility. These findings, interpreted considering retrospective constraints, warrant validation in larger cohorts.

## 1. Introduction

Fibrous dysplasia (FD) is a rare, benign skeletal disorder caused by postzygotic activating mutations in the *GNAS* gene [1,2]. This genetic alteration leads to constitutive activation of the Gαs signaling pathway, resulting in abnormal accumulation of cyclic AMP (cAMP) and disruption of osteoblastic differentiation [3,4]. Histologically, FD manifests as the replacement of normal bone architecture with immature fibro-osseous tissue, which progressively expands within medullary cavities [5]. Craniofacial involvement poses particularly complex challenges due to the intricate anatomy of this region; a substantial proportion of patients develop clinically significant complications including vision impairment, hearing loss, and severe disfigurement [5,6,7,8]. Surgical intervention remains the cornerstone of management, with conservative bone contouring widely adopted to restore function and esthetics [6,9]. However, the clinical course is frequently complicated by postoperative regrowth, with reported recurrence rates ranging from 32% to 82% in prior reports, depending on patient selection, anatomical site, and follow-up duration [10,11]. This high recurrence burden imposes substantial physical, psychological, and socioeconomic costs on patients [12,13,14,15], highlighting an urgent need for reliable prognostic tools to guide surgical decision-making.

Alkaline phosphatase (ALP), a well-established biochemical marker of osteoblastic activity, has emerged as a candidate biomarker for FD progression [16,17,18]. Its appeal stems from both biological plausibility—given FD’s pathophysiology of aberrant bone remodeling [3,19]—and clinical accessibility through routine blood testing. Nevertheless, the literature presents conflicting evidence regarding ALP’s prognostic utility. Some investigators have reported correlations between elevated ALP levels and disease recurrence, documenting abrupt enzymatic surges preceding radiographically confirmed regrowth [17,20]. Conversely, other well-designed studies found no significant differences in ALP profiles between patients with stable versus progressive lesions [11], suggesting that ALP elevation may reflect generalized bone turnover rather than specific disease activity [21]. This fundamental discrepancy underscores critical knowledge gaps in the field [9,13]. First, the influence of skeletal maturity thresholds on ALP dynamics remains poorly characterized, despite biological evidence that FD progression often stabilizes after puberty in many patients [9,15,22]. Second, the interaction between ALP and lesion-specific characteristics—particularly laterality (unilateral vs. bilateral) and disease subtype (monostotic, polyostotic, or craniofacial)—has not been systematically evaluated [5,23]. Third, prior studies have predominantly examined ALP in isolation [11,17,21], neglecting its potential integration with clinical variables for comprehensive risk stratification.

To address these questions, this retrospective cohort study pursued two primary objectives: First, to rigorously quantify associations between preoperative ALP levels and key clinical parameters including patient age at surgery, disease onset chronology, lesion laterality patterns, and pathological subtypes. Second, clinical factors associated with postoperative regrowth using χ^2^ or Fisher’s exact tests, specifically evaluating whether ALP retains prognostic value in relation to established clinical variables. By anchoring our investigation in the clinical paradoxes surrounding ALP while addressing specific mechanistic and methodological limitations of prior research [11,13], this work aims to refine prognostic frameworks for craniofacial FD management.

## 2. Materials and Methods

### 2.1. Patients

This retrospective study analyzed 71 consecutive patients with histopathologically confirmed fibrous dysplasia (FD) treated at Peking University Hospital of Stomatology between 2003 and 2024. Given the rarity of FD, this cohort represents all eligible cases during this period. Inclusion required documented preoperative serum alkaline phosphatase (ALP) levels using Stanley’s reference range [24] (Table 1), complete clinical records, and histopathological confirmation. Cases lacking ALP data or with incomplete records were excluded. Sixty-four patients (90.1%) completed ≥1-year follow-up.

FD diagnosis was established through integrated clinical examination, imaging, and histopathology. Cases were categorized as monostotic (single bone), polyostotic (≥2 non-contiguous bones), or craniofacial (multi-bone involvement crossing cranial sutures). Demographic characteristics, clinical symptoms, affected sites, and surgical details were systematically recorded.

Lesion activity was assessed during follow-up by two clinicians reviewing serial radiographs and clinical records. Active lesions exhibited confirmed radiographic progression with clinical regrowth, while stable lesions showed no significant changes.

This study received ethical approval from the Institutional Review Board of Peking University School of Stomatology (No. PKUSSIRB-2025113146), with waived informed consent for anonymized retrospective data analysis.

### 2.2. Statistical Analysis

All statistical analyses were performed using GraphPad Prism 10 and SPSS 27. Continuous variables with non-normal distribution were analyzed using the Mann–Whitney U test or Kruskal–Wallis test. Categorical variables were compared using the chi-square test, adjusted chi-square test, or Fisher’s exact test, depending on sample size and expected frequency. A *p*-value < 0.05 was considered statistically significant, with Bonferroni correction applied for multiple comparisons. All tests were two-sided.

## 3. Results

### 3.1. Cohort Characteristics and Clinical Phenotypes

This retrospective study analyzed 71 consecutive craniofacial fibrous dysplasia patients undergoing surgical management. As detailed in Table 2, the cohort demonstrated a balanced gender distribution: 46.5% males (33/71) and 53.5% females (38/71). Disease onset predominantly occurred during skeletal development, with 80.3% (57/71) presenting before age 16 years (median onset: 10.0 years). Surgical intervention was typically performed after skeletal maturity, as evidenced by 80.3% (57/71) undergoing procedures at >19 years (median surgical age: 25.0 years).

Lesion characteristics revealed distinct patterns: unilateral involvement predominated (73.2%, 52/71), while bilateral lesions affected 26.8% (19/71). Subtype distribution was nearly equivalent between monostotic (MFD, 39.4%, 28/71) and polyostotic (PFD, 39.4%, 28/71) forms, with craniofacial-specific disease (CFD) accounting for 21.1% (15/71).

A standardized surgical approach was implemented: conservative bone contouring in 70 patients (98.6%) versus osteotomy in one (1.4%). Progressive deformity represented the primary concern (95.8%, 68/71), while pain (12.7%, 9/71) and numbness (4.2%, 3/71) were less common. During a median 56-month follow-up, recurrence occurred in 8.5% (6/71) of patients, all requiring revision surgery.

### 3.2. ALP Associations with Disease Phenotypes

Analysis of preoperative alkaline phosphatase revealed significant variations across clinical phenotypes (Table 3 and Table 4). Adolescents undergoing surgery (16–19 years) exhibited substantially higher ALP levels than adults (median 244.0 vs. 107.0 U/L, *p* < 0.001) and greater abnormal ALP rates (*p* = 0.010). Similarly, patients with early disease onset (0–16 years) showed elevated ALP (median 114.0 vs. 83.0 U/L, *p* = 0.030) and increased ALP abnormality frequency (*p* = 0.018) compared to later-onset cases.

**Figure 1:**
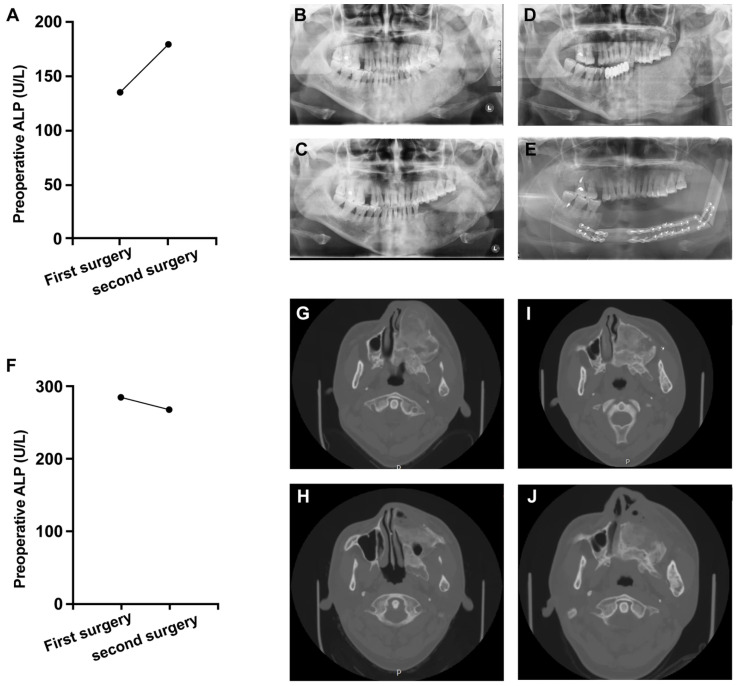
Two cases of patients with active lesion progression. (**A**) Preoperative ALP levels at the time of the first and second surgeries in a female patient with fibrous dysplasia in the bilateral mandible. (**B**) Preoperative panoramic radiograph from the first surgery showing a ground-glass, ill-defined mass in the mandible (Date: 12 August 2014). (**C**) Postoperative radiograph following the first surgery (Date: 29 August 2014). (**D**) Preoperative radiograph from the second surgery showing significant progression of the lesion compared to image (**C**) (Date: 4 September 2019). (**E**) Postoperative radiograph following the second surgery (Date: 16 September 2019). (**F**) Preoperative ALP levels at the time of the first and second surgeries in a male patient with unilateral polyostotic fibrous dysplasia in the left maxilla, sphenoid bone, ethmoid bone and left mandible. (**G**) Preoperative panoramic radiograph from the first surgery showing a ground-glass, ill-defined mass in the left craniofacial regions (Date: 12 March 2015). (**H**) Postoperative radiograph following the first surgery (Date: 17 April 2015). (**I**) Preoperative radiograph from the second surgery showing significant regrowth of the lesion compared to image (**H**) (Date: 18 May 2018). (**J**) Postoperative radiograph following the second surgery (Date: 25 May 2018).

Bilateral lesions demonstrated pronounced ALP elevation relative to unilateral involvement, with both higher median values (176.0 vs. 106.2 U/L, *p* = 0.006). Significant subtype variations emerged: polyostotic FD (PFD) displayed the highest ALP levels (median 162.0 U/L) and abnormality prevalence (66.7%), contrasting sharply with monostotic FD (MFD: 87.5 U/L, 10.0% abnormal; overall *p* < 0.001). Figure 1 shows two cases of active lesion progression: A female with MFD, after initial bone contouring, ALP rose from 135 U/L to 180 U/L with radiographic progression, requiring segmental resection. A male with PFD, after initial bone contouring, ALP decreased slightly (285 U/L to 268 U/L) but radiographic progression occurred, requiring repeat bone contouring.

Notably, neither ALP levels (active vs. stable: 236.5 vs. 107.0 U/L, *p* = 0.061) nor abnormal rates (16.7% vs. 38.5%, *p* = 0.090) significantly predicted regrowth. Gender differences showed no ALP correlation (*p* = 0.588).

### 3.3. Association Between Clinical Characteristics and Postoperative Disease Progression

Table 5 summarizes the associations between clinical characteristics and postoperative disease progression (active vs. stable lesions). Laterality was the only variable significantly associated with progression. Bilateral lesions accounted for 83.3% (5/6) of the active group compared with 21.5% (14/65) of the stable group (*p* = 0.005), whereas unilateral lesions predominated in the stable group (78.5% (51/65)) but were uncommon in the active group (16.7% (1/6)).

No significant associations were observed for the remaining variables. Age at surgery and age of onset were comparable between groups (median [IQR] age at surgery: 20.5 (17.3–27.5) vs. 23.0 (20.0–29.0) years, *p* = 0.336; age of onset: 7.0 (5.3–10.3) vs. 12.0 (9.0–15.0) years, *p* = 0.136). Sex distribution did not differ between groups (*p* = 0.543), nor did FD subtype (*p* = 0.426).

Regarding biochemical indicators, ALP-related variables showed a non-significant trend toward association with progression. The active group had a higher median ALP level (236.5 (151.1–381.8) U/L) than the stable group (107.0 (84.0–156.0) U/L), but this difference did not reach statistical significance (*p* = 0.061). Similarly, abnormal ALP status was more frequent in the active group (83.3%) than in the stable group (38.5%), without statistical significance (*p* = 0.090).

## 4. Discussion

FD lesions are often challenging to resect completely, and residual dysplastic tissue may predispose patients to postoperative regrowth, resulting in substantial economic and psychological burdens [9,12,13,22,25]. FD typically manifests in childhood and progresses through adolescence, with growth arrest commonly occurring after puberty. While surgery is often deferred until disease stabilization, some cases exhibit continued growth into adulthood [22], highlighting the unpredictable nature of FD progression and the urgent need for reliable prognostic markers.

Alkaline phosphatase (ALP), an important osteoblastic enzyme, is widely used as a prognostic biomarker for bone-related diseases [16,17,18]. However, its role in FD prognosis remains controversial [11,17,20,21]. Several studies have investigated the correlation between serum ALP levels and FD regrowth. Park et al. reported a significant relationship between local recurrence and postoperative ALP levels, observing an abrupt increase in ALP prior to craniofacial FD regrowth in seven patients, which was confirmed by CT imaging [17]. Hussein et al. similarly demonstrated a significant association between ALP levels and lesion regrowth during long-term follow-up [20]. Conversely, Ma et al. found no significant difference in ALP levels between patients with and without recurrence, suggesting that elevated preoperative ALP levels may be influenced by physiological bone metabolism rather than lesion activity. They proposed that increased ALP levels might be related to calcitonin-induced intracellular ALP activation via cAMP signaling [11]. Wang et al. further reported that preoperative ALP and calcium levels were not predictive of local recurrence but were associated with the progression of Shepherd’s crook deformity, highlighting the potential role of ALP monitoring in identifying deformity progression [21].

Our results demonstrated that preoperative ALP levels and abnormal rates were significantly associated with age at surgery, age of onset, laterality, and lesion type. However, preoperative ALP levels and abnormal rates were not significantly associated with regrowth. Patients undergoing surgery between 16 and 19 years of age exhibited significantly higher ALP levels and abnormal rates compared to those aged ≥19 years, likely due to higher bone turnover rates in younger individuals. Additionally, patients with an onset age of 0–16 years exhibited significantly higher ALP levels and abnormal rates than older patients, likely due to longer disease duration and sustained osteoblastic activity of the patients who were affected earlier. Notably, statistically significant differences in ALP levels and abnormal rates were observed between MFD and CFD, and MFD and PFD (*p* = 0.022; *p* < 0.001), which were partially consistent with previous studies [11,21].

To further investigate the prognostic risk factors, we identified a significant difference in laterality between patients with active and stable lesions. Thus, bilaterality was significantly associated with postoperative regrowth of postoperative regrowth, as illustrated in Figure 1, emphasizing the need for close monitoring in these patients. Furthermore, the observed overall regrowth rate of 8.5% was notably lower than rates reported in previous studies. This difference may be attributed to our patient population’s older surgical age (all patients ≥16 years), as skeletal maturity typically completes around this age, leading to more stable lesions at the time of intervention. In contrast, prior studies included younger patients who underwent surgery before complete skeletal maturation, which might explain their higher reported regrowth rates [17].

Nevertheless, several limitations must be acknowledged. First, the sample size was relatively small, with only six cases of regrowth, limiting the statistical power to fully elucidate the relationship between ALP and regrowth. The low event count may have reduced our ability to detect a moderate prognostic effect of ALP (i.e., a potential Type II error). Accordingly, the non-significant association should be interpreted with caution, and ALP should not be dismissed outright, as it may better reflect overall phenotypic burden rather than serve as a standalone predictor of regrowth. Second, because most patients underwent surgery after skeletal maturity, the observed regrowth rate may be lower than that reported in cohorts enriched for pediatric cases [17]. Therefore, generalizability to younger patients or those with rapidly progressive disease is limited, and prospective validation in age-diverse cohorts is needed to determine whether bilaterality remains predictive across disease stages. Third, lesion activity or regrowth assessment in our retrospective cohort relied on serial imaging reviewed by two clinicians in consensus together with clinical documentation of regrowth. Although this approach is clinically practical, it remains subject to some degree of observer variability, and objective quantification such as standardized CT-based volumetric analysis was not consistently feasible due to heterogeneous imaging protocols. Future prospective studies with harmonized imaging acquisition and volumetric endpoints are warranted to reduce subjectivity and improve comparability across cohorts [26,27]. Finally, other bone turnover markers, such as serum calcium, phosphate, and resorptive markers, were not assessed, which could provide additional insights into FD pathophysiology.

Our findings indicate that preoperative ALP levels were not significantly different between FD patients with and without regrowth, reinforcing its limitations as a standalone prognostic marker. Preliminary observations indicate that bilateral lesions may be associated with more active disease behavior. While further validation is needed, these findings suggest bilaterality might represent a potential risk factor for postoperative regrowth, highlighting the importance of vigilant monitoring in this patient subgroup. FD remains a lifelong condition requiring prolonged follow-up. Future studies with larger cohorts and comprehensive bone metabolism analyses are needed to validate these findings and further refine prognostic models for FD.

## Figures and Tables

**Table 1:** The reference values of ALP for children and adults.

Age	Value (U/L)	
**1–9 years**	145–420	
**10–11 years**	140–560	
	**Male**	**Female**
**12–13 years**	200–495	105–420
**14–15 years**	130–525	70–230
**16–19 years**	65–260	50–130
**>19 years**	45–125	50–135

**Table 2:** General characteristics of patients with FD between 2003 and 2024 (*n* = 71).

Items	*N*	Percent (%)
**Age of onset**		
0–16 years	57	80.3
>16 years	14	19.7
**Gender**		
Male	33	46.5
Female	38	53.5
**Age at surgery**		
16–19 years	14	19.7
>19 years	57	80.3
**Types of surgical procedures**		
Conservative bone contouring	70	98.6
Osteotomy	1	1.4
**Laterality**		
Unilateral lesions	52	73.2
Bilateral lesions	19	26.8
**Type**		
MFD	28	39.4
CFD	15	21.2
PFD	28	39.4
**Symptoms**		
Deformity	68	95.8
Pain	9	12.7
Numbness	3	4.2
**Prognosis**		
Active lesions	6	8.5
Stable lesions	65	91.5

**Table 3:** Analysis of the clinical characteristics for preoperative ALP of patients with FD.

Items	*N* (%)	Preoperative ALP	*p*-Value
**Age at surgery**			**0.001**
16–19 years	14 (19.72)	244.00 (137.50, 411.25)	
>19 years	57 (80.28)	107.00 (79.46, 144.00)	
**Age of onset**			**0.030**
0–16 years	57 (80.28)	114.00 (88.08, 200.00)	
>16 years	14 (19.72)	82.97 (61.92, 129.62)	
**Gender**			0.588
Male	33 (46.48)	114.00 (84.00, 167.00)	
Female	38 (53.52)	109.62 (85.00, 160.69)	
**Laterality**			**0.006**
Unilateral lesions	52 (73.24)	106.15 (78.54, 140.73)	
Bilateral lesions	19 (26.76)	176.00 (112.08, 364.46)	
**Type**			**<0.001**
MFD	28 (39.44)	87.46 (72.77, 107.00)	
CFD	15 (21.13)	115.15 (88.19, 200.31)	
PFD	28 (39.44)	162.00 (113.98, 286.15)	
**Prognosis**			0.061
Active lesions	6 (8.45)	236.50 (151.13, 381.75)	
Stable lesions	65 (91.55)	107.00 (84.00, 156.00)	

*p*-value < 0.05 was considered statistically significant and bolded.

**Table 4:** Evaluation of the rate of abnormal preoperative ALP for patients with FD.

Items	*N* (%)	ALP Normal No.	ALP Abnormal No.	*p*-Value
**Age at surgery**	23.00 (20.00, 29.00)	25.00 (22.00, 31.00)	21.50 (18.00, 25.75)	**0.010**
**Age of onset**	12.00 (8.00, 15.00)	13.00 (10.00, 16.00)	10.00 (6.25, 12.75)	**0.018**
**Gender**				0.322
Male	33 (46.48)	17 (41.46)	16 (53.33)	
Female	38 (53.52)	24 (58.54)	14 (46.67)	
**Laterality**				**0.031**
Unilateral lesions	52 (73.24)	34 (82.93)	18 (60.00)	
Bilateral lesions	19 (26.76)	7 (17.07)	12 (40.00)	
**Type**				**<0.001**
MFD	28 (39.44)	25 (60.98)	3 (10.00)	
CFD	15 (21.13)	8 (19.51)	7 (23.33)	
PFD	28 (39.44)	8 (19.51)	20 (66.67)	
**Prognosis**				0.090
Active lesions	6 (8.45)	1 (2.44)	5 (16.67)	
Stable lesions	65 (91.55)	40 (97.56)	25 (83.33)	

*p*-value < 0.05 was considered statistically significant and bolded.

**Table 5:** Analysis of the clinical characteristics for prognosis for FD patients.

Items	*N* (%)	Active Lesions	Stable Lesions	*p*-Value
**Age at surgery**	23.00 (20.00, 29.00)	20.50 (17.25, 27.50)	23.00 (20.00, 29.00)	0.336
**Age of onset**	12.00 (8.00, 15.00)	7.00 (5.25, 10.25)	12.00 (9.00, 15.00)	0.136
**Gender**				0.543
Male	33 (46.48)	4 (66.67)	29 (44.62)	
Female	38 (53.52)	2 (33.33)	36 (55.38)	
**Laterality**				**0.005**
Unilateral lesions	52 (73.24)	1 (16.67)	51 (78.46)	
Bilateral lesions	19 (26.76)	5 (83.33)	14 (21.54)	
**Type**				0.426
MFD	28 (39.44)	1 (16.67)	27 (41.54)	
CFD	15 (21.13)	1 (16.67)	14 (21.54)	
PFD	28 (39.44)	4 (66.67)	24 (36.92)	
**ALP status**				0.090
ALP normal no.	41 (57.75)	1 (16.67)	40 (61.54)	
ALP abnormal no.	30 (42.25)	5 (83.33)	25 (38.46)	
**ALP levels**	110.23 (84.19, 166.31)	236.50 (151.13, 381.75)	107.00 (84.00, 156.00)	0.061

*p*-value < 0.05 was considered statistically significant and bolded.

## Data Availability

The data presented in this study are available on request from the corresponding author due to ethical reasons.

## References

[1] Hartley I., Zhadina M., Collins M.T., Boyce A.M. (2019). Fibrous Dysplasia of Bone and McCune-Albright Syndrome: A Bench to Bedside Review. Calcif. Tissue Int..

[2] Riminucci M., Robey P.G., Saggio I., Bianco P. (2010). Skeletal progenitors and the GNAS gene: Fibrous dysplasia of bone read through stem cells. J. Mol. Endocrinol..

[3] Bianco P., Riminucci M., Majolagbe A., Kuznetsov S.A., Collins M.T., Mankani M.H., Corsi A., Bone H.G., Wientroub S., Spiegel A.M. (2000). Mutations of the GNAS1 gene, stromal cell dysfunction, and osteomalacic changes in non-McCune-Albright fibrous dysplasia of bone. J. Bone Miner. Res..

[4] Boyce A.M., Florenzano P., de Castro L.F., Collins M.T. (2019). Fibrous Dysplasia/Mccune-Albright Syndrome.

[5] Margaret Flanagan A., Fitzpatrick S., Nelson B.L. (2022). Fibrous Dysplasia. Head and Neck Tumours.

[6] Szymczuk V., Taylor J., Boyce A.M. (2023). Craniofacial Fibrous Dysplasia: Clinical and Therapeutic Implications. Curr. Osteoporos. Rep..

[7] Pereira T.D.F., Gomes C.C., Brennan P.A., Fonseca F.P., Gomez R.S. (2019). Fibrous dysplasia of the jaws: Integrating molecular pathogenesis with clinical, radiological, and histopathological features. J. Oral Pathol. Med..

[8] WHO (2013). WHO Classification of Tumours of Soft Tissue and Bone: WHO Classification of Tumours.

[9] Lee J.S., FitzGibbon E.J., Chen Y.R., Kim H.J., Lustig L.R., Akintoye S.O., Collins M., Kaban L. (2012). Clinical guidelines for the management of craniofacial fibrous dysplasia. Orphanet J. Rare Dis..

[10] Kusano T., Hirabayashi S., Eguchi T., Sugawara Y. (2009). Treatment strategies for fibrous dysplasia. J. Craniofacial Surg..

[11] Ma J., Liang L., Gu B., Zhang H., Wen W., Liu H. (2013). A retrospective study on craniofacial fibrous dysplasia: Preoperative serum alkaline phosphatase as a prognostic marker?. J. Cranio-Maxillofac. Surg..

[12] Pan K.S., Taylor J., Szymczuk V., Boyce A.M. (2023). Lesion Expansion in Gnathic Fibrous Dysplasia: Natural History, Indicators of Progression, and Response to Bisphosphonates. J. Bone Miner. Res..

[13] Javaid M.K., Boyce A., Appelman-Dijkstra N., Ong J., Defabianis P., Offiah A., Arundel P., Shaw N., Pos V.D., Underhil A. (2019). Best practice management guidelines for fibrous dysplasia/McCune-Albright syndrome: A consensus statement from the FD/MAS international consortium. Orphanet J. Rare Dis..

[14] Chen P.R., Chuang K.T., Wang P.F., Yao C.F., Chou P.Y., Chen Y.A., Lin C.C.H., Huang H.Y., Chen Y.R. (2026). Craniofacial fibrous dysplasia: Long-term postoperative outcomes in a retrospective case series with up to 40 years of follow-up. Bone.

[15] Sweeney K., Kaban L.B. (2020). Natural History and Progression of Craniofacial Fibrous Dysplasia: A Retrospective Evaluation of 114 Patients from Massachusetts General Hospital. J. Oral Maxillofac. Surg..

[16] Sadeghi S.M., Hosseini S.N. (2011). Spontaneous conversion of fibrous dysplasia into osteosarcoma. J. Craniofacial Surg..

[17] Park B.Y., Cheon Y.W., Kim Y.O., Pae N.S., Lee W.J. (2010). Prognosis for craniofacial fibrous dysplasia after incomplete resection: Age and serum alkaline phosphatase. Int. J. Oral Maxillofac. Surg..

[18] Coleman R., Brown J., Terpos E., Lipton A., Smith M.R., Cook R., Major P. (2008). Bone markers and their prognostic value in metastatic bone disease: Clinical evidence and future directions. Cancer Treat. Rev..

[19] Meier M.E., Hagelstein-Rotman M., Streefland T.C.M., Winter E.M., Bravenboer N., Appelman-Dijkstra N.M. (2023). Clinical value of RANKL, OPG, IL-6 and sclerostin as biomarkers for fibrous dysplasia/McCune-Albright syndrome. Bone.

[20] Hussein M.A., Yun I.S., Kim B.O., Kim Y.O. (2017). Craniofacial Fibrous Dysplasia: Retrospective Study on the Relationship Between the Tumor Volume Changes and the Circulating Serum Calcitonin and Serum Alkaline Phosphatase. Ann. Plast. Surg..

[21] Wang J., Du Z., Li D., Yang R., Tang X., Yan T., Guo W. (2020). Increasing serum alkaline phosphatase is associated with bone deformity progression for patients with polyostotic fibrous dysplasia. J. Orthop. Surg. Res..

[22] Yamagishi Y., Okamoto M., Yoshimura Y., Kito M., Aoki K., Takahashi J. (2020). Continued growth of locally aggressive fibrous dysplasia of 22 years duration after reaching adulthood: A case report. J. Surg. Case Rep..

[23] Burke A.B., Collins M.T., Boyce A.M. (2017). Fibrous dysplasia of bone: Craniofacial and dental implications. Oral Dis..

[24] Lo S.F., Roper S.M., Robert M.M.D., Kliegman R.M., Blum N.J., Tasker R.C., Wilson K.M., Schuh A.M., Mack C.L., Deardoff M.A. (2025). Nelson Textbook of Pediatrics.

[25] Boyce A.M., Burke A., Cutler Peck C., DuFresne C.R., Lee J.S., Collins M.T. (2016). Surgical Management of Polyostotic Craniofacial Fibrous Dysplasia: Long-Term Outcomes and Predictors for Postoperative Regrowth. Plast. Reconstr. Surg..

[26] Kim Y.C., Han S.J., Choi J.W. (2024). Functional outcomes and recurrence determinants in craniofacial fibrous dysplasia: Insights from 3D computed tomography and comprehensive clinical evaluation. J. Plast. Reconstr. Aesthetic Surg..

[27] Li G., Liu H., Pan Z., Cheng L., Dai J. (2025). Predicting craniofacial fibrous dysplasia growth status: An exploratory study of a hybrid radiomics and deep learning model based on computed tomography images. Oral Surg. Oral Med. Oral Pathol. Oral Radiol..

